# Supplementary material for: Quantitative Investigation of FAD2 Cosuppression Reveals RDR6-Dependent and RDR6-Independent Gene Silencing Pathways
Source: Int J Mol Sci. 2023 Dec 6;24(24):17165. doi: 10.3390/ijms242417165 (PMC10742939; doi:10.3390/ijms242417165)
Supplement: Supplementary file 1 [file ijms-24-17165-s001.zip › ijms-2725028-supplementary.pdf]

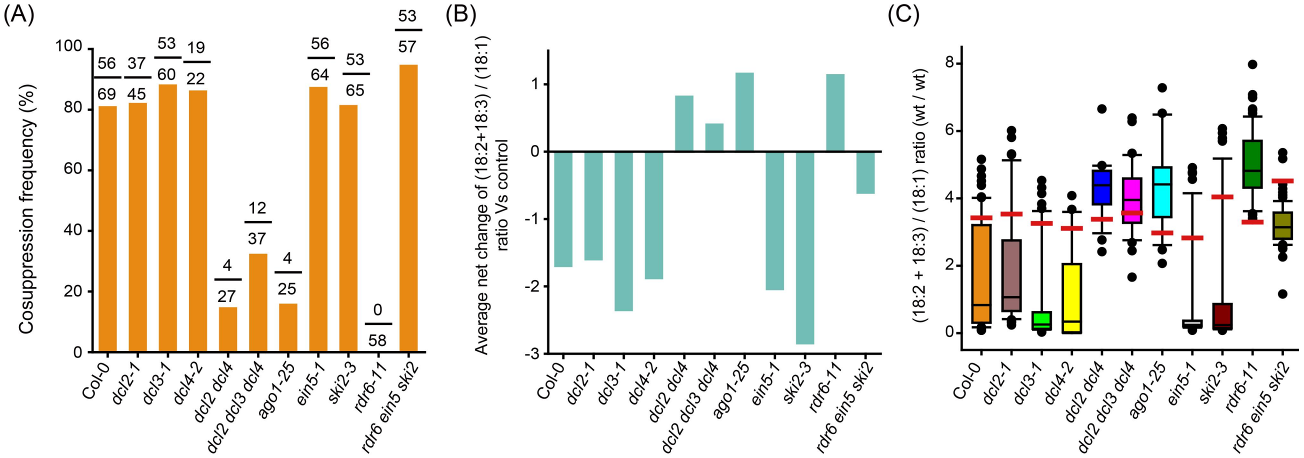

Figure S1. *FAD2* cosuppression frequencies and average net change in (18:2+18:3)/18:1 ratios in T_1 seeds in wild type or the designated mutant backgrounds.

(A) Cosuppression frequencies, the lines with a lower average (18:2+18:3)/18:1 ratio than their respective host controls are considered as cosuppression lines.

(B) Average net change of (18:2+18:3)/ 18:1 ratios in T_1 seeds in wild type or the designated mutant backgrounds. Net change refers to the difference in average fatty acid proportions between transgenic seeds and wild type Col-0 or corresponding mutant seeds. The average net change refers to the average net change of all lines detected.

(C) Boxplots showing the distribution of (18:2+18:3)/ 18:1 ratios in T_1 seeds in wild type or the designated mutant backgrounds. The red bars represent values of (18:2 + 18:3)/18:1 ratio in wild type Col-0 seeds and corresponding mutant seeds.

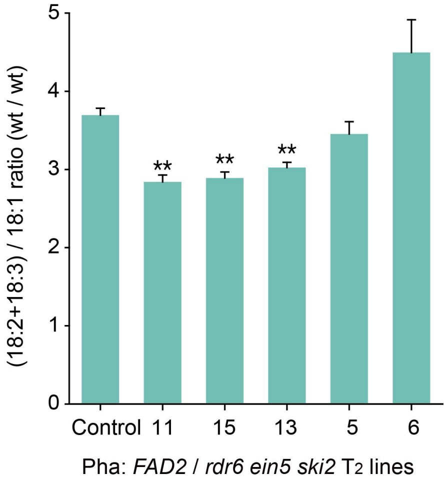

Figure S2. Ratios of (18:2+18:3)/18:1 proportions in seeds of wild type and T_2 Pha::*FAD2*/*rdr6 ein5 ski2* lines.
Pha::*FAD2*/*rdr6 ein5 ski2* refers to *FAD2*, driven by the Phaseolin promoter, transferred to the triple *rdr6 ein5 ski2*. Error bars indicate SD (n = 3). Ten T_2 seeds with red fluorescence are used in each replicate. Asterisk (**) indicates a significant difference (t-test, P < 0.01)

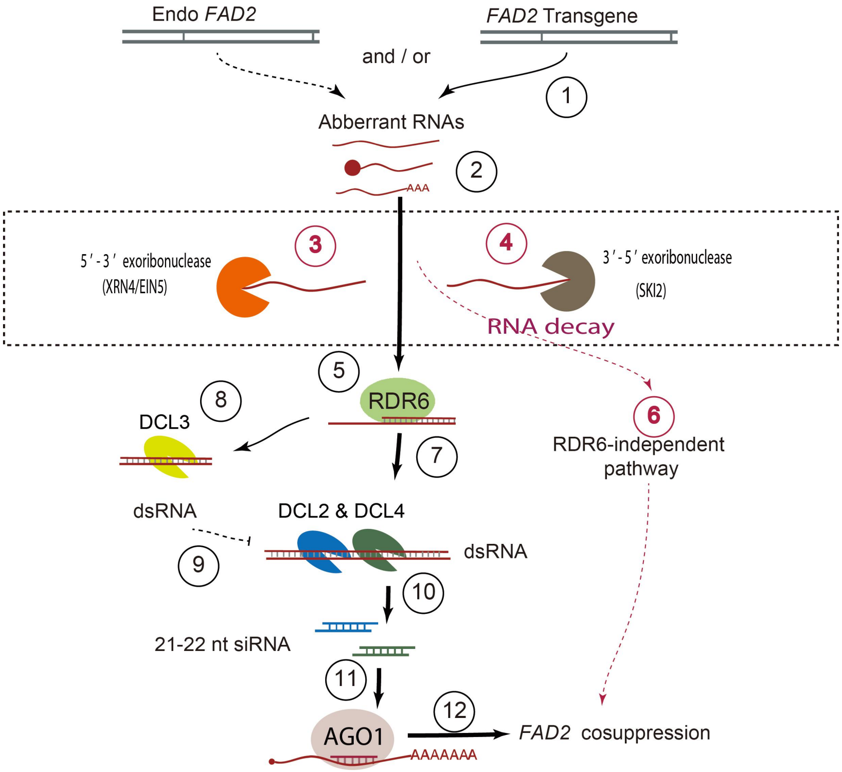

Figure S3. Suggested mechanistic pathway for *FAD2* cosuppression.
(1) The aberrant RNA triggered by transgene transcription. However, it is uncertain whether active aberrant RNAs are produced from the endogenous gene.
(2) The active aberrant RNAs are deduced to be incomplete RNA without 5’ cap or 3’ tail, or both. This hypothesis is supported by the fact that *FAD2* cosuppression is inhibited by EIN5 or SKI2, which degrades aberrant RNA without a 5’ cap or 3’ tail, respectively.
(3) and (4) RNA decay system are involved in *FAD2* cosuppression.
(5) Aberrant RNAs, devoid of degradation by the cytoplasmic RNA decay system, are converted to double-strand RNAs by RDR6. Here, *FAD2* cosuppression is also mediated by RDR6.
(6) A potential mechanism of *FAD2* cosuppression is independent of RDR6. The weak cosuppression fatty acid phenotype observed in Pha::*FAD2*/*rdr6 ein5 ski2* lines suggest that the mechanism is inhibited by EIN5 and/or SKI2 and results in a weaker silencing than that created by the RDR6-mediated mechanism.
(7) Double-strand RNAs, generated by RDR6, are processed into 21-22 nt siRNAs by DCl2 and DCl4. *FAD2* cosuppression depends on the presence of the two dicers, DCL2 and DCL4. Either the DCL2 or DCL4 mutation alone, could not fully release *FAD2* cosuppression.
(8) and (9) Considering DCL3 could convert double-strand RNA from RDR6 into small RNAs, inhibition of PTGS by DCL3 may occur through completion with substrates of DCL2 and DCL4.
(10) and (11) 21 and 22 nt siRNAs, generated by DCL2 and DCL4 respectively, result in *FAD2* cosuppression as they account for 80% of the siRNA pool present in cosuppressionors. More importantly, dysfunction of DCl2 and DCl4 release *FAD2* cosuppression
(12) AGO1 binds siRNAs and slices the target mRNA (*FAD2* in this study).
